# Catechol-*O*-Methyltransferase (COMT) Protein Expression and Activity after Dopaminergic and Noradrenergic Lesions of the Rat Brain

**DOI:** 10.1371/journal.pone.0061392

**Published:** 2013-04-16

**Authors:** Nadia Schendzielorz, Juha-Pekka Oinas, Timo T. Myöhänen, Ilkka Reenilä, Atso Raasmaja, Pekka T. Männistö

**Affiliations:** Division of Pharmacology & Toxicology, Faculty of Pharmacy, University of Helsinki, Helsinki, Finland; Karolinska Inst, Sweden

## Abstract

The occurrence of catechol-O-methyltransferase (COMT) in presynaptic neurons remains controversial. This study utilized dopaminergic and noradrenergic toxins to assess the presence of COMT in the presynaptic neurons originating from the substantia nigra, ventral tegmental area or locus coeruleus. Destruction of dopaminergic and noradrenergic neurons was assessed by measuring the dopamine and noradrenaline content in the projection areas of these neurons. Additionally, COMT protein expression and activity were examined in several projection areas to determine whether there are any changes in COMT values. Colocalization studies were done to identify COMT-containing postsynaptic neurons. Despite successful lesioning of dopaminergic and noradrenergic neurons, no changes in COMT protein expression or activity could be noted. These results strongly suggest that COMT is not present in presynaptic dopaminergic and noradrenergic neurons. There was a high colocalization of COMT with the GABAergic marker of short neurons both in the striatum and cortex but only a weak, if any, with the cholinergic marker in the cortex.

## Introduction

In mammals, catechol-O-methyltransferase (COMT) O-methylates catechol containing compounds, like catecholamines, L-dopa, catecholestrogens and dietary polyphenols [1]. COMT is widely distributed throughout the body with highest activities observed in peripheral tissues, particularly in the liver, kidney and gut [1]. The localization of COMT in the brain still remains controversial. COMT is an intracellular enzyme that exists in two isoforms: a membrane-bound (MB-COMT) and a soluble form (S-COMT). Neither of the two isoforms is present in the extracellular fluid nor the plasma membrane [1]. S-COMT resides in the cytoplasm and nuclei while MB-COMT is associated with the rough endoplasmic reticulum [1]. Along with immunohistochemical studies, a number of lesion studies concluded that COMT does not reside in presynaptic dopaminergic neurons and is present only at low levels in postsynaptic neurons [2]; [3]; [4]. Instead, high levels of COMT protein were found in non-neuronal cells, i.e. in ependymal cells of the cerebral ventricles, choroid plexus and glial cells [3]; [5]; [6]; [7]. Interestingly, a recent study by Matsumoto and colleagues [8] detected higher COMT mRNA levels in neurons than in non-neuronal cells in the prefrontal cortex and striatum, in both human and rats. However, COMT mRNA levels have previously been shown to poorly correlate with actual COMT protein levels [9]. In contrast to previous reports, Chen and colleagues [10] recently proposed that MB-COMT is present in presynaptic neurons and that its catalytic domain is oriented towards the extracellular space in a primary culture of rat cortical neurons.

This study investigated the localization of COMT protein in dopaminergic and noradrenergic terminals of the rat brain. Selective lesions of dopaminergic cell bodies were made by injecting 6-hydroxydopamine (6-OHDA) site-specifically to the lateral substantia nigra (SN) or ventral tegmental area (VTA). Noradrenergic neurons originating from the locus coeruleus were damaged by intraperitoneal (i.p.) injection of N-(2-chloroethyl)-N-bromobenzylamine hydrochloride (DSP-4) [11]. COMT protein expression and enzyme activity were measured in the intact and lesioned projection areas to assess the presence of COMT in the distinct presynaptic dopaminergic and noradrenergic sites. Moreover, colocalization of COMT with GABAergic and cholinergic neuronal markers were studied using double-label immunofluorescence.

## Materials and Methods

### Chemicals

All chemicals used were purchased from Sigma-Aldrich (St. Louis, MO, USA) unless an alternative source is specified in the text. Ethanol was purchased from Altia (Helsinki, Finland).

### Ethics Statement

Male adult Wistar rats (body weights between 265–360 g in 6-OHDA studies and 210–240 g in DSP-4 studies) were obtained from Harlan, The Netherlands. Rats were housed in clear polycarbonate cages in groups of 2–4. All rats were maintained under a 12∶12 h light/dark cycle with lights on from 06∶00 to 18∶00 at an ambient temperature of 20–22°C. Standard rat chow and tap water were available *ad libitum*. The experiments were conducted according to the “European Convention for the protection of Vertebrate Animals used for Experimental and other Scientific purposes”, and reviewed and approved by the National Animal Experiment Board.

### Treatment Groups

Rats were divided into 3 groups: group 1 received unilateral infusions of 6-OHDA to the lateral SN (n = 15), group 2 received unilateral infusions of 6-OHDA to the VTA (n = 15) and group 3 received i.p. injections of DSP-4 (n = 20) or saline (n = 20). To prevent noradrenergic neurons from taking up 6-OHDA, rats received i.p. injections of desipramine (25 mg/kg) 30 min prior to infusion of 6-OHDA. Desipramine effectively protects noradrenergic neurons in 6-OHDA treated rats [12]. As we only infused 6-OHDA unilaterally, the intact side of the brain was used as a control value to decrease the number of experimental animals. In earlier studies we have not seen any differences when using either the intact side or the intact sham-operated controls. Animals were sacrificed 3 weeks (6-OHDA groups) or 2 weeks (DSP groups) post lesion and COMT protein expression and activity were measured from respective projection areas of the lateral SN, VTA or locus coeruleus.

### 6-OHDA Lesions of the Lateral SN

**Figure 1:**
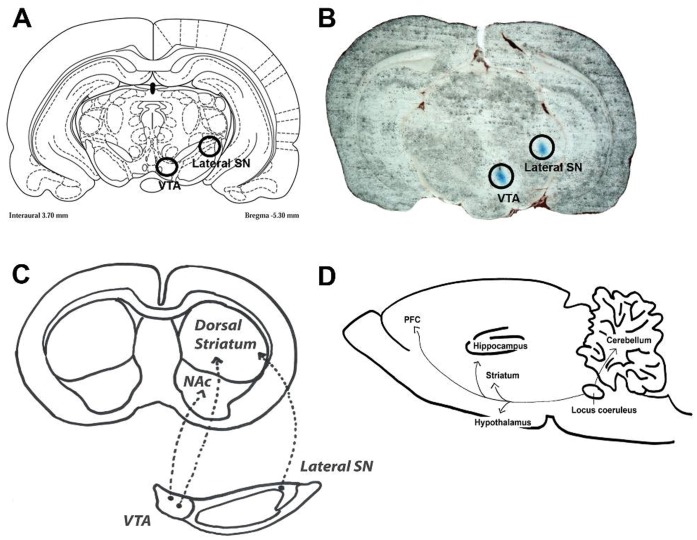
Illustration of the injection sites in dopaminergic lesions in the rat brain. (A) Anatomical locations of the two injection sites in the Paxinos Rat Atlas (circles). (B) Actual tissue slice demonstrating the positions of the two injection sites (blue colour). (C) Illustration of the main projection areas of the ventral tegmental area (VTA) and lateral substantia nigra (SN). (D) Projection areas of the locus coeruleus.

Stereotaxic surgery and injections of 6-OHDA were performed in four sessions under isoflurane anaesthesia (4.5% during induction and 2–3% during maintenance). A total amount of 8 µg of 6-OHDA (4 µg/µl, dissolved in 0.02% ascorbic acid) was unilaterally injected into the right lateral SN (A/P −5.3; L/M −3.0; D/V −6.6) at a flow rate of 0.5 µl/min using a 10 µl Hamilton microsyringe run by a microdialysis pump (QSI, Stoetling, Wood Dale, Il, USA). The coordinates were measured from bregma according to the rat brain atlas [13]. For an illustration of the injection site and main projection ares [14]; [15]; [16], please refer to Figure 1A–C.

### 6-OHDA Lesions of the VTA

As above, a total amount of 8 µg of 6-OHDA (4 µg/µl, dissolved in 0.02% ascorbic acid) was unilaterally injected into the right VTA (A/P −5.3; L/M −0.9; D/V −8.3) at a flow rate of 0.5 µl/min using a 10 µl Hamilton microsyringe run by a microdialysis pump (QSI, Stoetling, Wood Dale, Il, USA). For an illustration of the injection site and main projection areas [14]; [15]; [16], please refer to Figure 1A–C.

### DSP-4 Lesions of the Locus Coeruleus

Rats received intraperitoneal injections of N-(2-chloroethyl)-N-bromobenzylamine hydrochloride (DSP-4, 50 mg/kg). The dose of DSP-4 was weighed separately for each animal, dissolved in saline and injected immediately. Control animals received saline injections. For an illustration of the main projection areas of the locus coeruleus [17]; [18], please refer to Figure 1D.

### Tissue Collection

Two separate experiments were made using either dopaminergic 6-OHDA or noradrenergic DSP-4 lesions. At the end of both the experiments, the rats were decapitated, the brains were immediately removed, and the samples dissected slightly differently in the two experiments. In the 6-OHDA studies, the tissue samples were collected separately from both brain sides and the unlesioned side was used as a control. In the DSP-4 studies, a group of unlesioned rats was used as a control, and the tissue samples from both sides of the brain were combined. Samples of specific brain areas were dissected based on visual inspection, landmarks and coordinates measured from bregma. In the 6-OHDA experiments, the samples from the nucleus accumbens (punching) and dorsal striatum were dissected separately in order to precisely dissociate these areas to compare the effects of the lateral substantia nigra and VTA lesions. In the DSP-4 experiments, these striatal areas were not separated. After dissection, the tissue samples were weighed and placed in plastic microfuge tubes, frozen on dry ice and stored at −80°C until further assayed.

In the 6-OHDA experiments, the brain samples were collected in the following order: the PFC, nucleus accumbens, dorsal striatum and hippocampus. The brains were placed on the ice-cold glass plate. The PFC was separated from the front of the brain with a razor blade and coronally sectioned as follows: the ventral area to the rhinal fissure was discarded and vertical cuts at 1 mm from the midline were made on both sides. A three mm thick slice containing both nucleus accumbens and dorsal striatum was separated frontally from the brain at optic chiasma (−0.3 mm). The nucleus accumbens was punched out from the slice (a coronal punch diameter was 2 mm). The dorsal striatum was squeezed off based on its visual looks with forceps frontally of the nucleus accumbens. Both hippocampal areas were finally rolled out and peeled off from the rest of the brain using forceps.

In the DSP-4 experiments, the samples were collected in the following order: the PFC, hypothalamus, whole striatum, hippocampus and cerebellum. The brains were dissected on the ice-cold glass plate. Both sides of the PFC were cut with a razor blade as described above. A coronal cut of the brain was made at optic chiasma (−0.3 mm). The hypothalamus was dissected by cutting its borders and pinched out. Both striata were removed frontally from the cut based on its visual looks by forceps as one sample containing both dorsal parts and nucleus accumbens. The hippocampus was peeled off from both sides and the cerebellum was removed with forceps.

### Monoamine Analysis

Brain levels of dopamine, noradrenaline and 5-hydroxytryptamine (5-HT) were measured using high-performance liquid chromatography (HPLC) with electrochemical detection as described previously [19]. Briefly, brain samples were dissected and placed into eppendorf tubes and kept on dry ice until stored at −80°C. The samples were homogenized in 0.5 ml of homogenization solution consisting of 6 parts of 0.2 M HClO_4_ and 1 part of antioxidant solution containing oxalic acid in combination with acetic acid and L–cysteine. The homogenates were centrifuged at 20 800 *g* for 35 min at 4°C. Supernatants were removed to 9.5 ml Vivaspin® filter concentrators (10 000 MWCO PES; Vivascience AG, Hannover, Germany) and centrifuged at 8600 *g* at 4°C for 35 min. The column (Spherisorb® ODS2 3 µm, 4.6×100 mm^2^; Waters, Milford, MA, USA) was kept at 50°C with a column heater (Croco-Cil, Bordeaux, France). The mobile phase consisted of 0.1 M NaH_2_PO_4_ buffer, 350 mg/l of octane sulfonic acid, methanol (3–6.5%), and 450 mg/l EDTA. The pH of the mobile phase was set to 2.7 using H_3_PO_4_. The pump (ESA Model 582Solvent Delivery Module; ESA, Chelmsford, MA, USA) was equipped with a pulse dampener (SSI LP-21, Scientific Systems, State College, PA, USA) and the flow rate was 1 ml/min. Sixty µl of the filtrate were injected into the chromatographic system with a CMA/200 autoinjector (CMA, Stockholm, Sweden).

Monoamines were detected using ESA® CoulArray Electrode Array Detector (voltages gradually increased from +100 to +300 mV), and chromatograms were processed and concentrations calculated using CoulArray® for windows software® (ESA, Chelmsford, MA, USA). CoulArray detector has several coulometric cells in series. Each cell has unique voltage and the analytes are analysed by corresponding dominant cell according to calibration sample response. These voltages are suitable for analysis of all reducable/oxidable monoamines.

When analysing either the 6-OHDA or DSP-4 lesioned tissues, we have used HPLC conditions optimized for dopamine or noradrenaline analysis, respectively. In case of dopamine run, noradrenaline comes very rapidly in the solvent front, whereas in the case of noradrenaline run the retention time of dopamine slows down. Here, for practical reasons, noradrenaline was not analysed in dopamine runs.

The monoamine values are expressed as nanograms per milligram (ng/mg) wet weight of tissue. Dopamine levels in terminal areas were measured after 6-OHDA treatments and noradrenaline and 5-HT levels after DSP-4 treatment. The detection limit of all these monoamine (i.e. dopamine, noradrenaline and 5-HT) analytes was 0.1 ng. The signal to noise -ratio was 5. The analytical variation within the day was 2.2% for dopamine, 12.1% for noradrenaline and 3.4% for 5-HT (n = 3 duplicate measurements).

### COMT Protein Expression

COMT protein amounts were measured using standard Western Blotting procedures as described previously [20]; [21]. In short, the tissue samples were homogenized using a sonicator (Rinco Ultrasonics sonicator, Arbon, Switzerland) in 20 volumes of homogenization buffer (10 mM Na_2_HPO_4_, pH 7.4, containing 0.5 mM dithiothreitol). The homogenates were centrifuged at 890 g at +4°C for 10 min and the supernatants were collected. The samples were then diluted with Laemmli buffer so that 20 µg of total protein was loaded and electrophoresis was performed in a 12% SDS-polyacrylamide gel. Subsequently, samples were blotted onto Protran^®^ nitrocellulose transfer membrane (Schleicher & Schuell Bioscience GmbH, Dassel, Germany). After blocking nonspecific binding, the membranes were incubated with mouse anti-COMT monoclonal antibody overnight at +4°C (1∶5000; BD Bioscience Pharmingen, San Diego, CA, USA). Following this, the membranes were processed with goat anti-mouse secondary antibody (1∶2000; R&D Systems, Minneapolis, MN, USA) conjugated with horseradish peroxidase. Blots were visualized using chemiluminescent substrate (Thermo scientific, Rockford, IL, USA) and detected and quantified using GeneGnome chemiluminescent detector and corresponding software (SynGene, Synoptics LTD, UK). Beta actin was used as a loading control. S-COMT and MB-COMT were seen as separate bands and the densities of these bands were quantified.

### COMT Activity Analysis

The COMT enzyme activity assay was assessed using HPLC with electrochemical detection as described earlier [20]; [21] using the same homogenate as with Western Blot studies. In brief, the enzyme homogenate preparation was incubated at +37°C in 100 mM phosphate buffer (pH 7.4) containing 5 mM MgCl_2_, 200 µM S-adenosyl-L-methionine and 500 µM 3,4-dihydroxybenzoic acid. A high-performance liquid chromatographic (HPLC) system with electrochemical detection was used to analyze the reaction products, vanillic acid and isovanillic acid. The system consisted of a sample autoinjector (Jasco AS-2057, Tokyo, Japan), a pump (Merck Hitachi LaChrom L-7100, Tokyo, Japan), an RP-18 column (3 mm, 4.6×150 mm; Waters Spherisorb, Milford, MA, USA), a coulometric detector (ESA Coulochem model 5100A detector and a model 5014B cell; ESA Inc., Chelmsford, MA, USA; E1 detector potential was −0.32 V and that of E2 analytical detector +0.5 V) and an integrator (Shimadzu C-R5A; Shimadzu Corporation, Kyoto, Japan). The mobile phase consisted of 0.1 M Na_2_HPO_4_ (pH 3.3), 0.15 mM EDTA and 25% methanol; the flow-rate was 0.8 ml/min.

The protein concentrations of the samples were determined based on the bicinchoninic acid method using Pierce protein assay kit (Pierce Biotechnology, Rockford, IL, USA). COMT activity is expressed as picomoles vanillic acid formed in one min per mg of protein in the sample.

### Double-label Immunofluorescence

COMT, tyrosine hydroxylase (TH), choline acetyltransferase (ChAT) and glutamic acid decarboxylase (GAD)-immunofluorescences were revealed by modifying the protocol described in Myöhänen et al. [7]. Briefly, rats were deeply anesthetized using pentobarbital (Orion Pharma, Espoo, Finland) and then perfused using saline followed by 4% paraformaldehyde in PBS, pH 7.4. After perfusion, the rats were decapitated, brains were removed and stored at −70°C until sectioning into 40 µm free-floating cryosections with a microtome (Leica M3050, Leica Microsystems Inc., Wetzlar, Germany). Free-floating sections were first washed with PBS, boiled for 30 min at +80°C in citrate buffer (pH 6.0) for antigen retrieval and then washed 3×15 min in PBS. After that, the sections were incubated in blocking solution (10% normal goat serum in PBS containing 0.5% Triton X-100; Vector laboratories, Burlingame, CA, USA) for 30 min followed by an overnight incubation with each primary antibody (see details of primary antibodies in Table 1). After washing with PBS the sections were incubated with secondary antibody (dilutions 1∶500, Fluorescein conjugated goat anti-rabbit IgG, Product # 31635, Pierce Biotechnology) for 2 h. Double-immunofluorescence with COMT-antibody followed after PBS washes. The procedure was similar to the first staining, and COMT-immunocomplexes were detected using rabbit anti-mouse with Texas Red conjugate (dilution 1∶500, Product #31610, Pierce Biotechnology) as a secondary antibody. Vectashield with DAPI (Vector Laboratories) was used as a mounting medium to demonstrate the nuclei of the cells. Control stainings for immunohistochemistry were carried out with the omission of the primary antibodies. For all immunostainings, three separate stainings from different animals were done.

**Table 1:** Details of primary antibodies.

Antigen	COMT	Glutamic acid decarboxylase65/67 (GAD 65/67)	Choline acetyltransferase (ChAT)	Tyrosine-hydroxylase (TH)
**Marker for**	COMT protein	GABAergic cells	Cholinergic cells	Dopaminergic cells
**Species**	Mouse IgG monoclonal	Rabbit IgG	Rabbit IgG	Rabbit IgG
**Immunogen**	Mouse COMT aa. 26–141	Synthetic peptide. Sequence: K-DIDFLIEEIERLGQDL	Synthetic peptide from porcineChAT. Sequence: H-GLFSSYRLPGHT- QDTLVAQKSS-NH_2_	Denaturated tyrosine hydroxylase from rat
**Manufacturer**	BD Biosciences; San Diego,CA, USA	Millipore; Billerica, MA, USA	Millipore	Millipore
**Product #**	Product 611970	Product AB1511	Product AB5042	Product AB152
**Dilution used**	1∶400	1∶500	1∶500	1∶500
**Specificity reference**	Western blot [7]	Western blot; Manucfacturer’s datasheet	Preadsorption control, Westernblot [32]	Western blot; Manufacturer’s datasheet

Fluorescent double-labeled sections were analyzed and photographed using Leica TCS SP2 AOBS (Leica Microsystems) equipped with an argon-He/Ne laser mounted on an inverted Leica DM IRE2 microscope (Leica Microsystems). After capturing the images with an imaging device, confocal double-labeled immunofluorescence micrographs were merged using Adobe Photoshop CS2 software (version 9.0, Adobe Systems Incorporated). Only minor corrections to brightness and contrast were made.

### Semiquantitative Analysis of COMT and TH Staining after Lesions

After capturing the fluorescent photomicrographs of the immunostained sections, the images were analyzed using Bio-Rad QuantityOne 4.5.1 software (Bio-Rad Laboratories, Hercules, CA, USA) as described earlier [22]. In optical density (OD) analysis, representative area of brain structure was delineated with the box tool of the software. The background value of each area (unstained zone in the area, e.g. cell nucleus) was subtracted from raw data values of the same area. The average of intact brain structure OD values was set as 100% and corresponding lesioned brain structure was compared to that value. Altogether 2–3 sections/animal from 3 different animals were analyzed.

### Statistics

All values are expressed as means ± SEM. Statistical analyses for significant differences was performed with unpaired t-test using GraphPad Prism 5.0 program (GraphPad Software, Inc., San Diego, CA, USA). The criterion for statistical significance was p<0.05.

## Results

### Dopamine Levels in the Projection Areas of the Lateral SN after 6-OHDA Lesion

**Figure 2:**
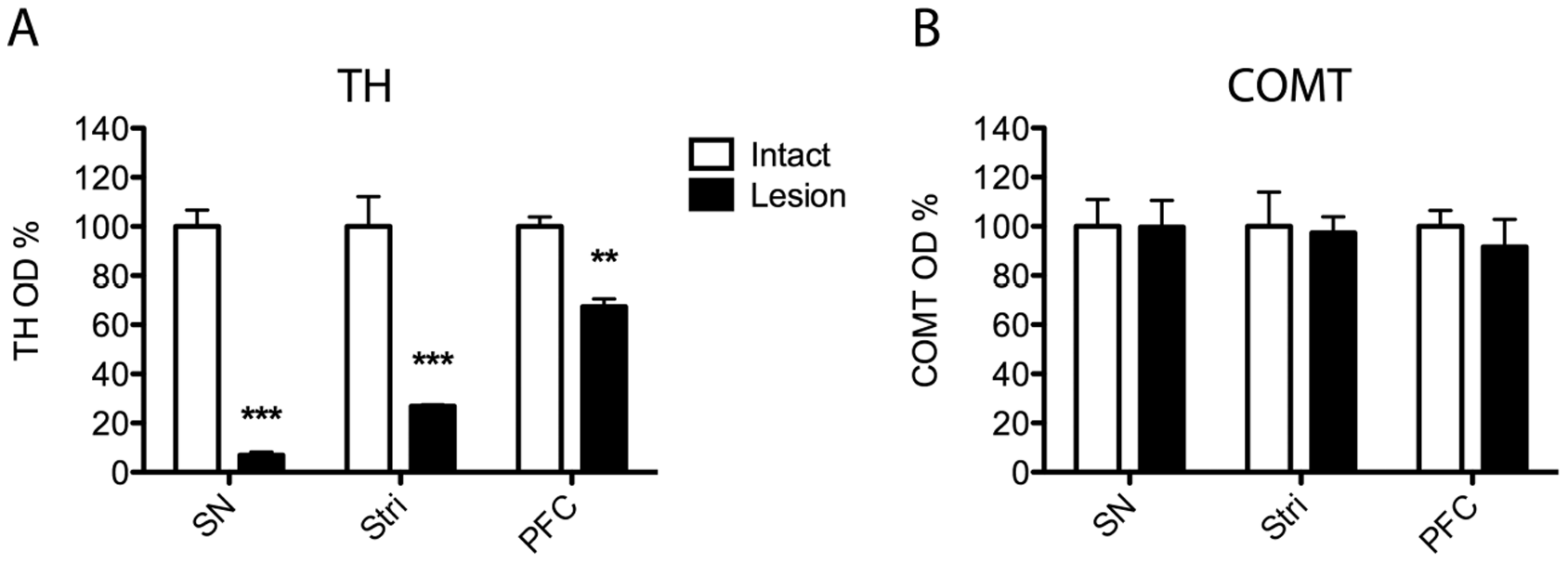
Successful dopaminergic lesions do not cause changes in the COMT protein levels. A normalized optical density (OD) analysis of (A) tyrosine hydroxylase (TH) or (B) COMT positive neurons in the substantia nigra (SN) and striatum (Stri) after a unilateral 6-OHDA (8 µg) lesion of the SN, and TH and COMT positive neurons in the prefrontal cortex (PFC) after 6-OHDA lesion of the VTA. At least 3 separate stainings were done, and mean ± SEM are shown. Significant decrease in TH OD was seen in all areas analyzed but none in COMT OD. ***, P<0.001; **, P<0.01 vs. control side (Student t-test).

Dopamine levels were decreased (p = 0.098; t = 1.870) to 56% in the dorsal striatum after selective lesion of the lateral SN (Table 2). In comparison, dopamine levels in other brain areas, e.g. the PFC and hippocampus (not shown) were not affected. Decreased dopamine levels in the dorsal striatum, the projection area of the lateral SN, indicate moderate but selective lesions of presynaptic nigrostriatal dopaminergic neurons. In a semiquantitative analysis, TH expression was significantly reduced both in the SN (to 7%) and striatum (to 27%; Fig. 2A).

**Table 2:** Dopamine levels after 6-OHDA-lesions and noradrenaline levels after DSP-4 lesions.

	Intact side or control animal		Lesioned side/animal	
6-OHDA lesion (lateral SN)	Dopamine (ng/mg)			
	n		N	
PFC	5	0.26±0.04	5	0.30±0.08 (116.9%)
Striatum	5	26.9±5.62	5	15.1±2.91 (56.1%)
NAcc	5	5.28±1.10	5	4.58±0.55 (86.7%)
Hippocampus	5	0.21±0.08	5	0.26±0.08 (123.8%)
**6-OHDA lesion (VTA)**	**Dopamine (ng/mg)**			
	**n**		**N**	
PFC	5	0.29±0.09	5	0.11±0.05 (38.2%)
Striatum	5	23.9±5.56	5	1.99±1.53 (8.3%)**
NAcc	5	6.3±1.88	3	0.68±0.28 (10.7%)*
Hippocampus	5	0.11±0.03	5	0.11±0.05 (93.1%)
**DSP-4 lesion**	**Noradrenaline (ng/mg)**			
	**n**		**N**	
PFC	5	0.682±0.059	4	0.029±0.006 (4.2%)***
Striatum	5	0.249±0.088	4	0.043±0.004 (17.5%)*
Hypothalamus	5	4642±0.307	4	2477±0.347 (53.4%)**
Hippocampus	5	0.582±0.045	4	0.063±0.06 (30.4%)***
Cerebellum	5	0.2495±0.088	4	0.009±0.004 (3.8%)*

The results (mean±sem) are expressed as absolute values (ng/mg) and as percentage (in parenthesis) of the intact side (6-OHDA) and control animals (DSP-4) set as 100. Significant differences from intact or controls values:

* p<0.05;

** p<0.01;

*** p<0.001. Key: PFC, prefrontal cortex; NAcc, nucleus accumbens; SN, substantia nigra; VTA, ventral tegmental area.

### Dopamine Levels in the Projection Areas of the VTA after 6-OHDA Lesion

Lesioning of the VTA with 6-OHDA resulted in decreased dopamine levels in the projection areas (Table 2), namely to about 38% in the PFC (p = 0.117; t = 1.176), to 8% in the dorsal striatum (p<0.01), and to 11% (p<0.05) in the nucleus accumbens. In the hippocampus, dopamine levels remained unaffected (not shown). In a semiquantitative analysis, TH expression was significantly reduced in the PFC (to 65%; Fig. 2A).

### Monoamine Levels in the Projection Areas of the Locus Coeruleus after DSP-4 Lesion

The locus coeruleus sends many projection neurons throughout the entire brain. We found decreased noradrenaline levels in the PFC (to 4%), striatum (to 18%), hypothalamus (to 53%) hippocampus (to 11%), and cerebellum (to 4%), indicating a successful destruction of noradrenergic neurons by DSP-4 (Table 2). 5-HT levels were in the control/lesioned animals as follow: 0.42±0.03/0.32±0.04 (PFC), 0.31±0.024/0.24±0.031 (striatum), 0.78±0.064/0.52±0.11 (hypothalamus), 0.27±0.059/0.12±0.014 (hippocampus) and 0.039±0.003/0.038±0.003 (cerebellum), respectively, showing a maximum decrease to 44% in the hippocampus (p = 0.228; t = 2.078). The unchanged dopamine levels (not shown) in any measured brain areas (e.g. 15.7 ng/mg and 14.8 ng/mg in the striatum of control (n = 5) and DSP-4 (n = 4) rats, respectively) from the DSP-4 treated rats support the selectivity of noradrenergic lesions.

### Expression of COMT and TH Proteins, and COMT Activity after 6-OHDA Infusion to the Lateral SN

**Figure 3:**
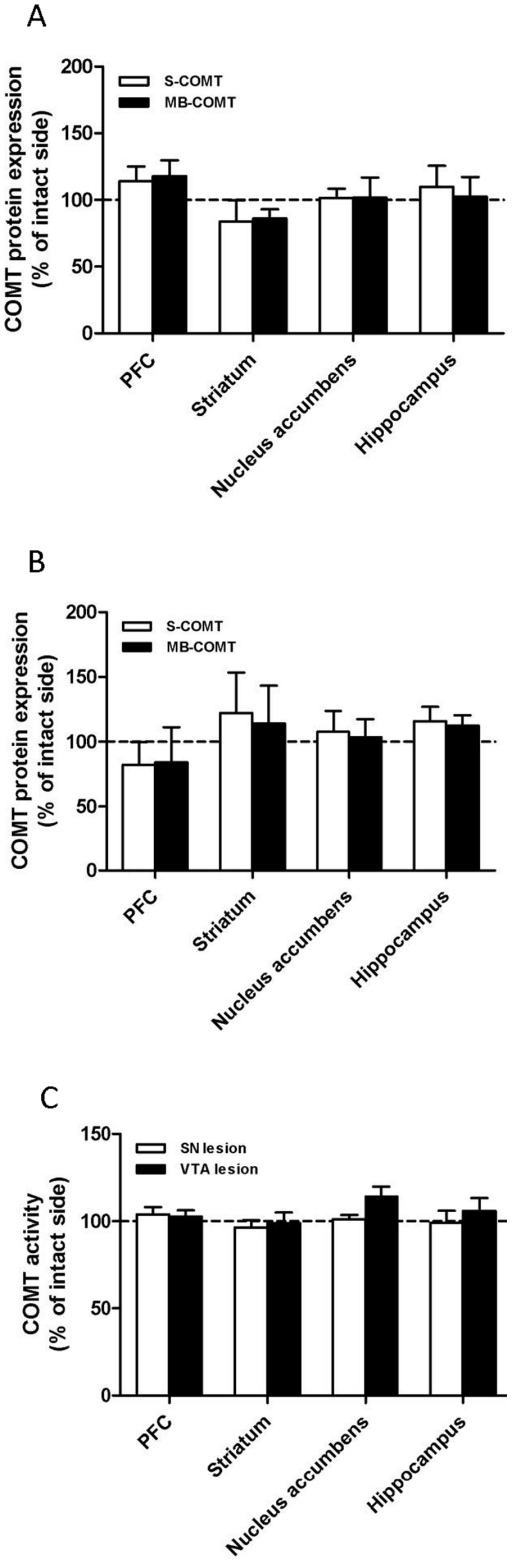
A–C. Selective dopaminergic lesions do not affect COMT protein expression and activity. 6-OHDA (8 µg) was unilaterally infused either to the lateral substantia nigra (SN) or ventral tegmental area (VTA). COMT protein expressions were unaltered in any of the dopaminergic projection areas in the SN (A) or VTA (B) in agreement with unchanged enzyme activity (C). In dopaminergic lesion studies, animals received unilateral infusions of 6-OHDA and therefore the results are expressed as percentage of the intact side set as 100. Mean ± SEM, n = 4 in the SN control group, otherwise n = 5.

**Figure 4:**
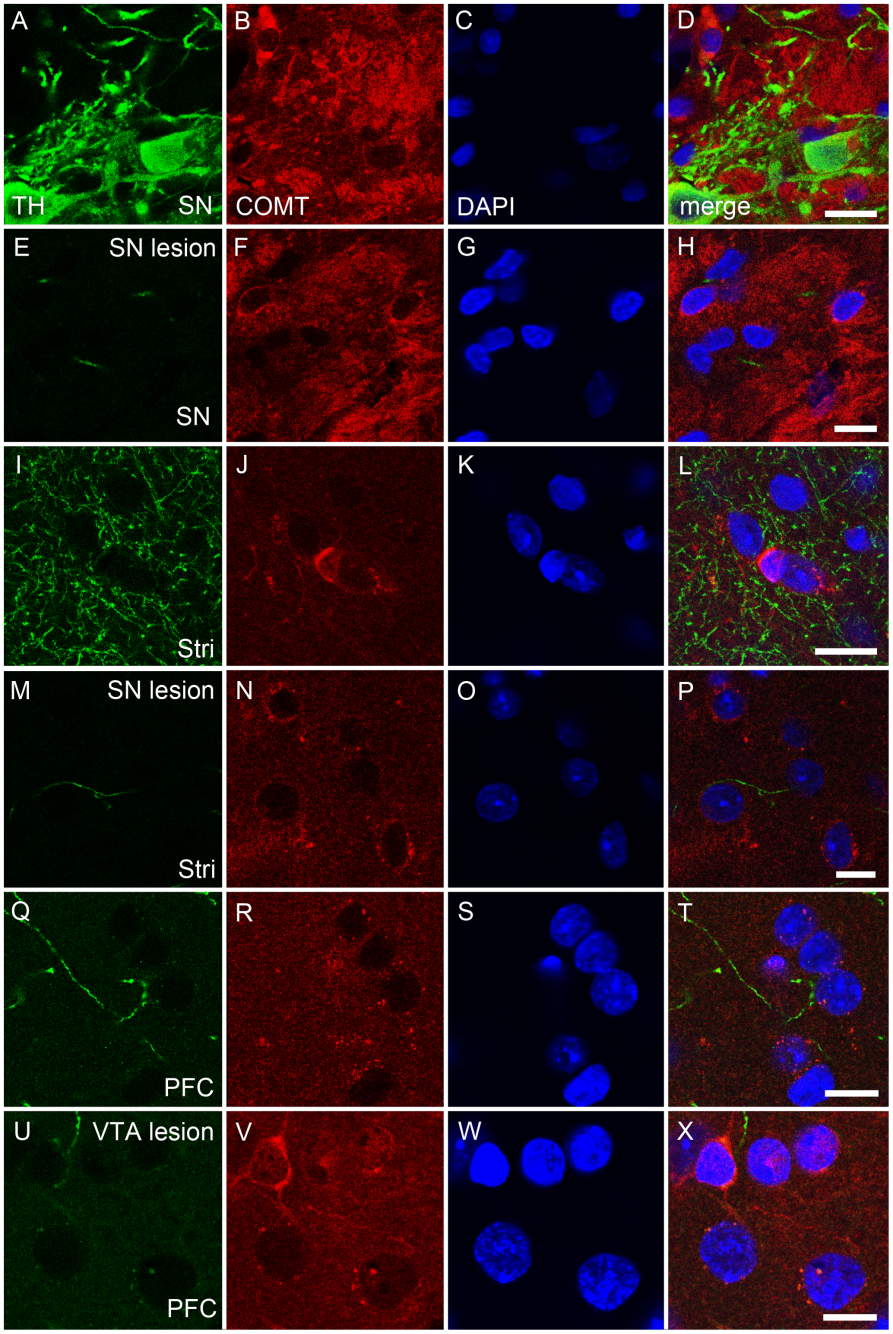
A–X. COMT protein neither colocalized with tyrosine hydroxylase (TH) positive neurons nor decreased after destruction of dopamine neurons. The effect of dopaminergic lesion on the COMT immunoreactivity. The 6-OHDA lesion of the substantia nigra (SN) and ventral tegmental area (VTA) were made as described and COMT immunoreactivity analysed in the projection areas. In the intact SN, COMT (red color) was not colocalized with dopaminergic neurons (tyrosine hydroxylase, TH; green color; A–D), and lesion in the SN did not affect COMT immunoreactivity (E–H) although TH-immunoreactivity disappeared (E). The situation was similar in the striatum (Stri; I–L versus M–P). Moreover, in the intact prefrontal cortex (PFC), the projection area of the VTA, COMT did not colocalize with dopaminergic nerves (Q–T). Moreover, the lesion in the VTA did not effect on COMT on the PFC (U–W). Nuclei are visualized by DAPI (blue color). Scale bars are 10 µm in all pictures. Three separate stainings were made and representative examples are shown. Quantification of ODs was done in Fig.2.

Despite successful lesioning of dopaminergic neurons projecting from the lateral SN to the dorsal striatum, expression of both COMT protein isoforms (Fig. 3A) and COMT activity (Fig. 3C) remained unchanged. These results indicate that COMT is not located in dopaminergic nigrostriatal projection neurons. Moreover, no significant changes in COMT-immunoreactivity was seen in the SN and striatum (Figs. 2B and 4A–P), although TH positive neurons disappeared from both brain regions after nigral lesions as a further proof of the dopaminergic damage (Fig. 2A).

### Expression of COMT and TH Proteins, and COMT Activity after 6-OHDA Infusion to the VTA

When dopaminergic neurons originating in the VTA and projecting to the nucleus accumbens or dorsal striatum were selectively destroyed by infusing 6-OHDA to the VTA, expression of both COMT protein isoforms (Fig. 3B) and COMT activity (Fig. 3C; e.g., for NAcc, p = 0.104, t = 1.836 ) remained unaltered, suggesting that COMT is not present in these neurons. This was verified by immunofluorescence staining, where no changes in the COMT-immunoreactivity was seen in the PFC (Figs. 2B and 4U–W), a projection area of the VTA although TH expression was strongly reduced supporting a significant dopaminergic damage (Figs. 2A and 4Q–X).

### COMT Activity and Expression of COMT and TH Proteins after DSP-4 Lesion

**Figure 5:**
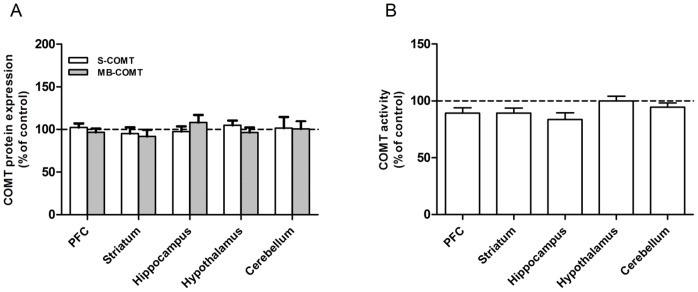
A–B. Selective noradrenergic lesions do not change COMT protein expression and activity. COMT protein expression (A) and activity (B) were unaltered in any of the noradrenergic projection areas. To destroy noradrenergic neurons, animals were injected i.p. with DSP-4 (50 mg/kg) and control animals received i.p. injections of saline (vehicle). The results are expressed as percentage of the control animals set as 100. Mean ± SEM, n = 5.

**Figure 6:**
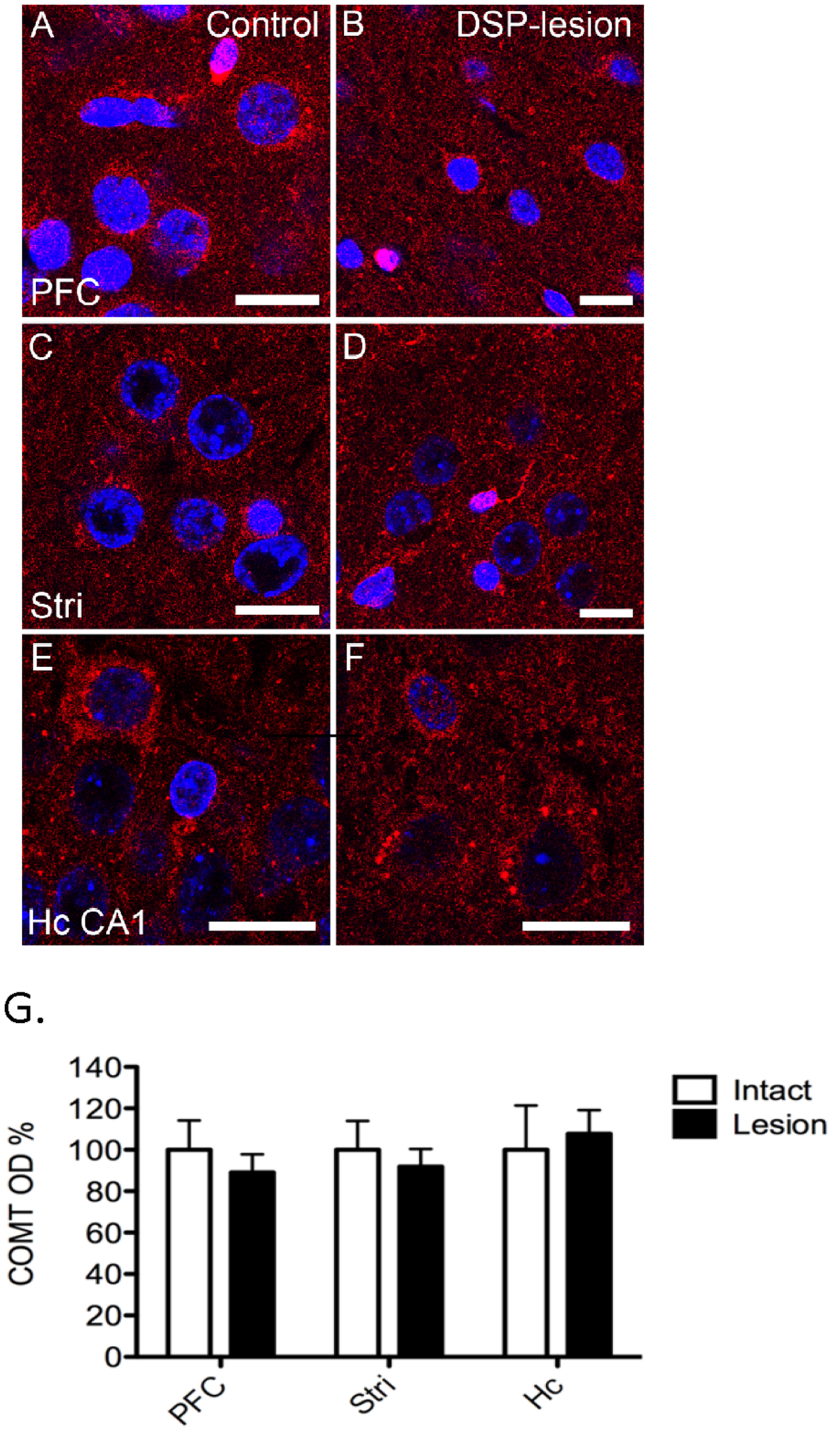
A–G. Lack of the effect of noradrenergic lesion on COMT immunoreactivity. DSP-4 lesion of noradrenergic neurons did not affect COMT-immunoreactivity (red color) in noradrenergic projection areas, the prefrontal cortex (PFC; A–B), striatum (Stri; C–D) and hippocampus CA1 (Hc CA1; E–F). Three separate stainings were done, and the COMT optical densities are quantified in Fig. 6G. Nuclei are visualized by DAPI (blue color). Scale bars are 10 µm in all pictures.

COMT protein expression (Fig. 5A) and COMT activity (Fig. 5B) were not affected by DSP-4 treatment. Furthermore, COMT immunoreactivity was not changed in the PFC, striatum or hippocampus (Fig. 6A–F). This observation suggests that COMT is not present in noradrenergic neurons originating from the locus coeruleus.

### Colocalization of COMT with GABAergic and Cholinergic Markers

**Figure 7:**
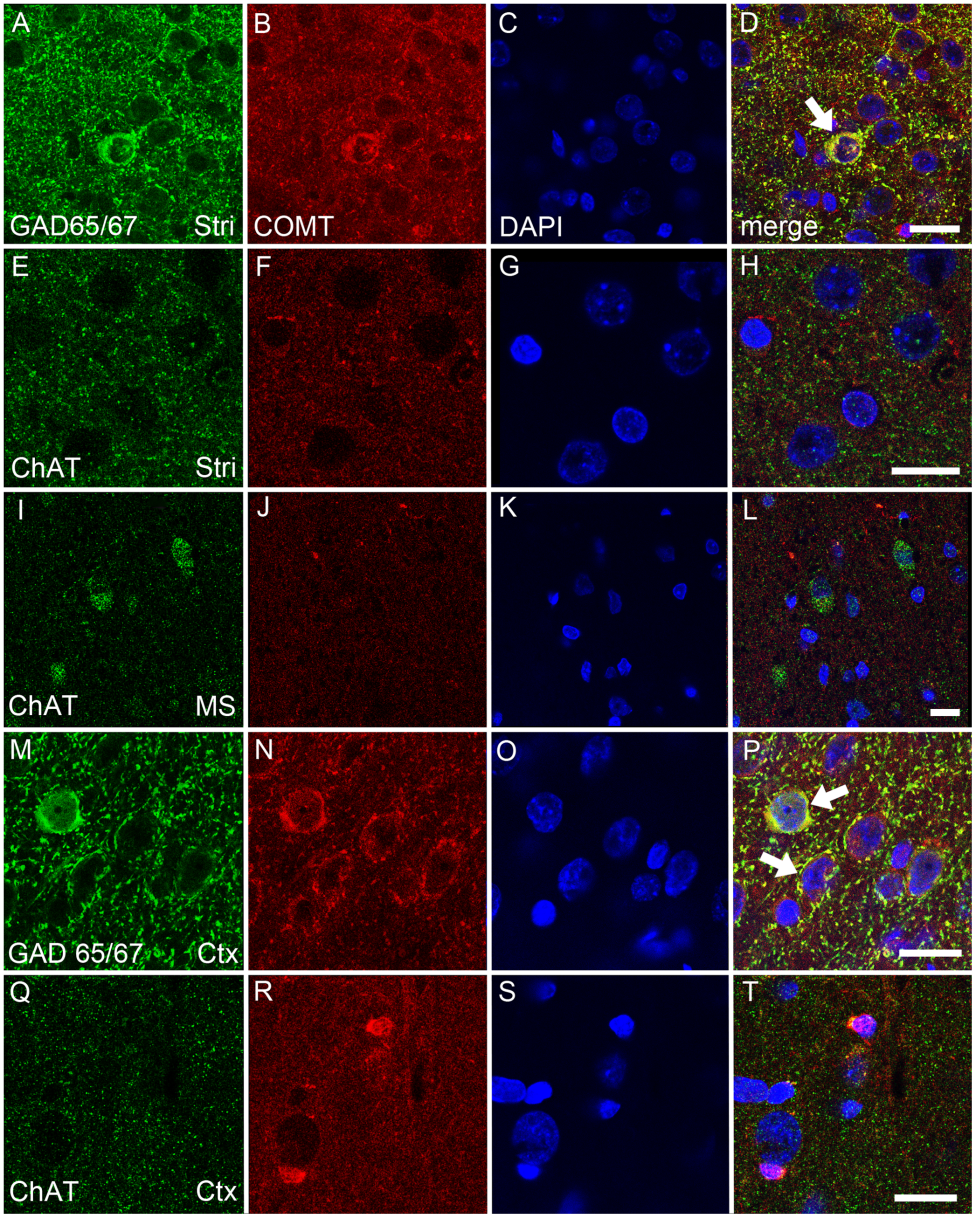
A–T. COMT protein is colocalized with a GABAergic (glutamate decarboxylase, GAD65/67; green) but less or none with a cholinergic marker (choline acetyltransferase, ChAT; green). In the striatum (Stri), COMT (red) was found to be colocalized with GABAergic neurons and fibres (A–D; white arrows and yellow color depicting colocalization), while no colocalization with cholinergic nerves was seen (E–H). Moreover, COMT was not colocalized with cholinergic neurons in the medial septum (MS; I–L). In the cortex, COMT was also colocalized with GABAergic interneurons (M–P; white arrows, yellow color) and a weak colocalization with some cholinergic nerves was seen (Q–T). Nuclei are visualized by DAPI (blue color). Scale bars are 10 µm in all pictures. Three separate stainings were done and representative examples are shown.

We also examined the colocalization of COMT with a GABAergic marker (GAD65/67) and a cholinergic marker (ChAT) in the striatum and cerebral cortex (primary motor cortex) in the DSP study. Moreover, a colocalization of COMT with ChAT was studied in the medial septum, one origin of cholinergic cell bodies [23]. COMT was seen colocalized with GABAergic neurons and nerve fibers in the striatum and cortex (Fig. 7A–D, M–P). Colocalization of COMT with cholinergic nerves was seen neither in the striatum (Fig. 7E–H) nor in cholinergic cell bodies in the medial septum (Fig. 7I–L). However, a weak colocalisation of COMT with cholinergic nerves in the cortex was observed (Fig. 7 Q–T).

## Discussion

The aim of this study was 1) to site-selectively destroy dopaminergic cell bodies located either in the lateral SN or VTA utilizing 6-OHDA, and 2) to demolish a major part of noradrenergic neurons via DSP-4, and 3) to subsequently measure potential changes in COMT activity and protein expression in several projection areas of the destroyed neurons. In addition, we also wanted to address the possible colocalization of COMT with GABAergic and cholinergic neurons in the respective projection areas using double-label immunofluorescence.

The presence of COMT in the nigrostriatal dopaminergic neurons has been previously investigated by Kaakkola and colleagues [2]. The authors lesioned the medial forebrain bundle which contains dopaminergic neurons originating from the VTA and SN and report unaltered COMT protein expression and activity in the striatum, a main target area. However, due to the extensive lesion the authors were unable to distinguish between the SN and VTA dopaminergic neurons. Our study was designed to selectively destroy either dopaminergic neurons originating from the SN or VTA, or noradrenergic neurons originating from the locus coeruleus, and to give a more thorough and detailed analysis of COMT enzyme in dopaminergic and noradrenergic projection areas. Because the SN and VTA are anatomically close to each other, we first confirmed that we are able to target either one of the two areas separately without damaging the adjacent area. It was confirmed that the diffusion patterns of methylene blue do not overlap in these two sites (Fig. 1B).

Successful lesioning of dopaminergic neurons originating from the lateral SN was confirmed by decreased dopamine levels in the dorsal striatum (to 56% compared to the intact side), the main projection area of the lateral SN. Moreover, in immunofluorescence, a disappearance of TH-positive dopaminergic neurons in the SN and striatum was observed. In comparison, dopamine levels remained unaltered in the PFC and hippocampus. The rather small decrease in the striatal dopamine is likely due to the non-optimum position of the lesion site. We purposely targeted only the lateral part of the SN to avoid spill-over of 6-OHDA to the VTA. Nevertheless, slightly decreased dopamine levels in the nucleus accumbens (to 87% compared to the intact side) suggest that some of the 6-OHDA may have spread to the medial part of the SN, since this area connects to the nucleus accumbens. Despite successful lesioning, the absence of changes in COMT protein and activity indicates that COMT is not present in dopaminergic neurons of the nigrostriatal tract. This was further supported by a double-label immunofluorescence, where no colocalization between COMT and TH-positive dopaminergic neurons was seen in the SN and striatum.

Decreased dopamine levels in the PFC (to 38% compared to the intact side), dorsal striatum (to 8% compared to the intact side) and nucleus accumbens (to 11% compared to the intact side) indicate an effective loss of dopaminergic neurons originating from the VTA. However, COMT protein and activity did not change. Collectively, these results strongly suggest that COMT is not localized in dopaminergic connections of the VTA.

Studies utilizing kainic acid to selectively destroy GABAergic and cholinergic neuronal cell bodies in the striatum report decreased MB-COMT activity, indicating the presence of MB-COMT in these postsynaptic terminals [4]. Furthermore, the authors described an increase in S-COMT activity, suggesting that S-COMT is present in glial cells [4]. An administration of 6-OHDA directly to the striatum causes a significant inflammatory response in the striatum and SN, and an increase in microglial response [24]. Several studies report similar findings after local insults [6]; [25], and that the increase in S-COMT is more pronounced than that of MB-COMT [25]. However, after the nigral injections of 6-OHDA to destroy nigrostriatal dopamine nerves, the glial response in the striatum has been scanty and absent within three weeks [26]; [27]. Moreover, COMT-immunoreactivity is even reduced in astroglial cells following insults [26]. Collectively, we assume that changes in the neuronal COMT activity and expression in the striatum, measured at 3 weeks post lesions of the SN, are not notably masked by a transient increase of microglial COMT in the striatum.

While the presence of COMT in presynaptic dopaminergic neurons has been extensively studied, only few reports address the existence of COMT in presynaptic terminals of noradrenergic neurons. Several studies propose that COMT is present in peripheral noradrenergic neurons [28]; [29]; [30]. However, the presence of COMT in presynaptic noradrenergic neurons in the brain is still uncertain [28]; [31]. Accordingly, in our study, although noradrenaline levels were strongly reduced in all analyzed projection areas of the locus coeruleus indicating effective lesions, we observed no changes in COMT protein expression or activity. These results indicate the absence of COMT from presynaptic noradrenergic neurons originating from the locus coeruleus. A selectivity of noradrenergic lesions was estimated by the measurement of 5-HT levels in the projection areas of the locus coereleus. After the DSP-4 lesions, the levels of noradrenaline were decreased significantly with minor decreases in 5-HT levels. Therefore, these results indicate that the obtained selectivity although not complete was sufficient for the present study.

Furthermore, when using a double-label immunofluorescence to examine the localization of COMT in GABAergic and cholinergic neurons in their projection areas [32]; [23], we detected the COMT protein especially in GABAergic neurons of the striatum and cortex. However, no convincing colocalization with a cholinergic marker, ChAT, was seen in cholinergic nerves or cell bodies of the striatum or septum. Only in some cortical areas a weak colocalization of COMT and cholinergic staining was seen, the latter in the nerve endings. These results show that, in addition to glial cells, COMT is localized in GABAergic neurons but evidently usually not in cholinergic neurons. Although we did not mark glutamatergic cells, we have shown before that COMT resides also in several brain areas that have glutamate as a major transmitter [7]. Therefore, it is probable that even the glutamatergic neurons contain COMT.

In conclusion, our results suggest that COMT is not significantly present in terminals of presynaptic dopaminergic and noradrenergic neurons. Instead, COMT is abundantly found in postsynaptic GABAergic neurons in the striatum and cortex.
